# Prognostic significance of autophagy-related genes within esophageal carcinoma

**DOI:** 10.1186/s12885-020-07303-4

**Published:** 2020-08-24

**Authors:** Chongxiang Chen, Siliang Chen, Huijiao Cao, Jiaojiao Wang, Tianmeng Wen, Xiaochun Hu, Huan Li

**Affiliations:** 1 Department of Intensive Care Unit, State Key Laboratory of Oncology in South China; Collaborative Innovation Center for Cancer Medicine, Sun Yat-sen University Cancer Center, 651 Dongfeng Dong Road, Guangzhou, 510060 People’s Republic of China; 2 Guangzhou Institute of Respiratory Diseases, State Key Laboratory of Respiratory Disease, the First Affiliated Hospital of Guangzhou Medical University, Guangzhou, China; 3 Department of hematology, State Key Laboratory of Oncology in South China; Collaborative Innovation Center for Cancer Medicine, Sun Yat-sen University Cancer Center, Guangzhou, China; 4 Department of VIP Inpatient, State Key Laboratory of Oncology in South China; Collaborative Innovation Center for Cancer Medicine, Sun Yat-sen University Cancer Center, Guangzhou, China; 5 Department of Tuberculosis, Fuzhou Pulmonary Hospital of Fujian, Fuzhou, China; 6 School of Public Health, Sun Yat-sen University, Guangzhou, China

**Keywords:** Esophageal carcinoma, Autophagy, Prognostic

## Abstract

**Background:**

Several works suggest the importance of autophagy during esophageal carcinoma development. The aim of the study is to construct a scoring system according to the expression profiles of major autophagy-related genes (ARGs) among esophageal carcinoma cases.

**Methods:**

The Cancer Genome Atlas was employed to obtain the esophageal carcinoma data. Thereafter, the online database Oncolnc (http://www.oncolnc.org/) was employed to verify the accuracy of our results. According to our results, the included ARGs were related to overall survival (OS).

**Results:**

We detected the expression patterns of ARG within esophageal carcinoma and normal esophageal tissues. In addition, we identified the autophagy related gene set, including 14 genes displaying remarkable significance in predicting the esophageal carcinoma prognosis. The cox regression results showed that, 7 ARGs (including TBK1, ATG5, HSP90AB1, VAMP7, DNAJB1, GABARAPL2, and MAP2K7) were screened to calculate the ARGs scores. Typically, patients with higher ARGs scores were associated with poorer OS. Moreover, the receiver operating characteristic (ROC) curve analysis suggested that, ARGs accurately distinguished the healthy people from esophageal carcinoma patients, with the area under curve (AUC) value of > 0.6.

**Conclusion:**

A scoring system is constructed in this study based on the main ARGs, which accurately predicts the outcomes for esophageal carcinoma.

## Background

Nowadays, studies around the world show that, esophageal carcinoma ranks the 7th and 6th places in terms of its morbidity tumor-related mortality cause [1]. However, the area distribution is imbalanced between cases and deaths, and some areas are regarded as the “esophageal carcinoma belt” [2, 3]. Several factors, such as smoking, obesity, low vegetable consumption, have been proven to adversely predict esophageal carcinoma [4, 5]. Nowadays, an increasing obesity trend in western countries results in the further increased esophageal carcinoma morbidity [1], as proven in one meta-analysis [6].

So far, several prognostic studies have used various indicators (including PET-CT [7], HER2 [8], Microsatellite instability [9] and PD-L1 expression [10]) to predict the long-term outcomes, and other studies have evaluated the treatment efficacy, or complications among esophageal carcinoma patients [11, 12]. Autophagy, a crucial biological process, balances the homeostasis in cells through the degradation of injured or aged organelles and proteins within lysosomes [13, 14]. Previous studies have validated the role of autophagy in promoting tumor cell survival and suppressing oncogenesis [15–17]. On this account, both enhancing and inhibiting autophagy have been suggested as the treatment strategies [18–21], which suggest that patient assessment plays an important role in autophagy. A large number of studies demonstrate that autophagy activation shows marked correlation with the tumor dormancy, chemoresistance, as well as stem cell survival [22].

According to previous studies, autophagy is correlated with esophageal carcinoma diagnosis and treatment [23]. However, the role of autophagy in the prognosis for esophageal carcinoma has scarcely been assessed by large-scale expression data. Therefore, this study was designed to construct a novel scoring system based on the screened important ARGs, which might contribute to a perspective tool in evaluating patient prognosis.

## Methods

### Autophagy related gene set

We identified autophagy related genes in human autophagy database.

### Patient samples

Both clinical data and gene expression patterns of esophageal carcinoma were retrieved based on The Cancer Genome Atlas (TCGA) database. Ultimately, a total of 171 specimens were collected based on TCGA microarray to be the cohort.

### Processes

First, we carried out Principal component analysis (PCA) using R program to investigate those different expression profiles of genes in the enrolled specimens. Second, we plotted the receiver-operating characteristics (ROC) curves using the survival ROC of R package to assess the survival specificity and sensitivity. Then, we determined the values of area under the ROC curve (AUC) based on those plotted ROC curves. Last, we used the online database Oncolnc to prove the accuracy of our results.

### Statistical analyses

Multivariate and univariate Cox regression analyses were carried out to assesse the correlation among ARGs, the risk score value (based on ARGs) and other clinical characteristics in prognosis prediction. Based on the risk score, patients with esophageal carcinoma were divided into low- and high-risk group according to the median score. The results of survival analyses were recognized as the key outcomes; and Kaplan-Meier analysis was used to analysis the patient prognosis (OS) among various ranges of score. The time-dependent ROC curves were plotted to calculate the risk model predictive ability. R software (version 3.6.1) was used to make Bioinformatic analyses. *P* < 0.05 was regarded as statistically significant. *X*^2^ test or Student’s t-test was used to analysis the differences of clinical baseline characteristics between low-risk and high-risk group.

## Results

### Differentially expressed ARGs (DEARGs) within esophageal carcinoma tissues

**Fig. 1:**
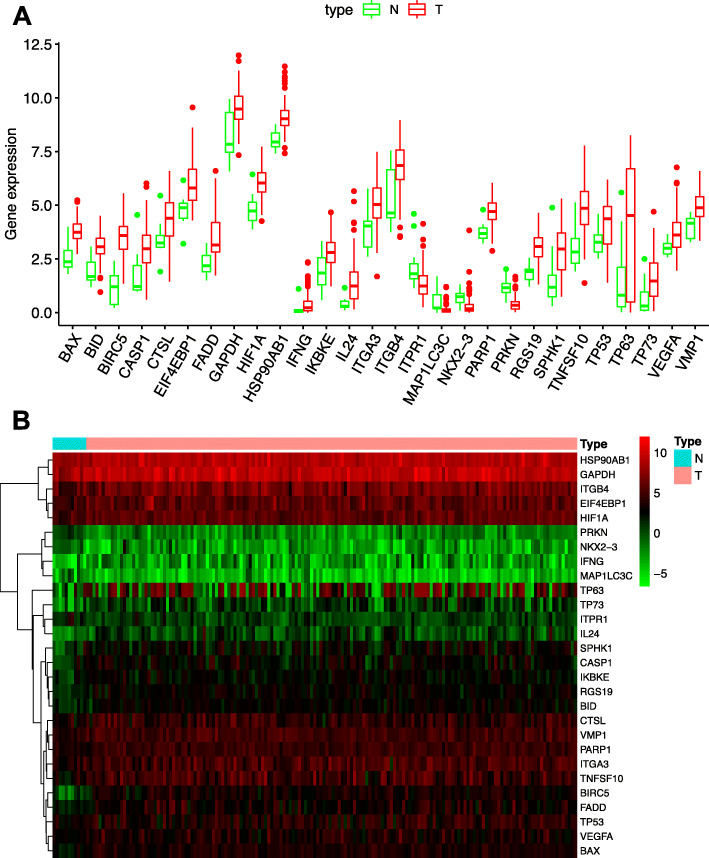
DEARGs within esophageal carcinoma tissues

Wilcoxon signed-rank test was carried out to analyze the 232 ARGs expression within 11 normal esophageal and 160 esophageal carcinoma samples, and 28 DEARGs were found, including 24 up-regulated and 4 down-regulated ones (|log2FC| > 1, FDR < 0.05, Fig. 1). Figure 1a was box plot of 28 DEARGs (red boxes showed the genic expression of tumor sample, and green boxes indicated the genic expression of normal sample); and Fig. 1b was the heat map of 28 DEARGs (red indicated higher expression and green indicated lower expression).

### Prognostic factor of DEARGs among esophageal carcinoma patients

**Fig. 2:**
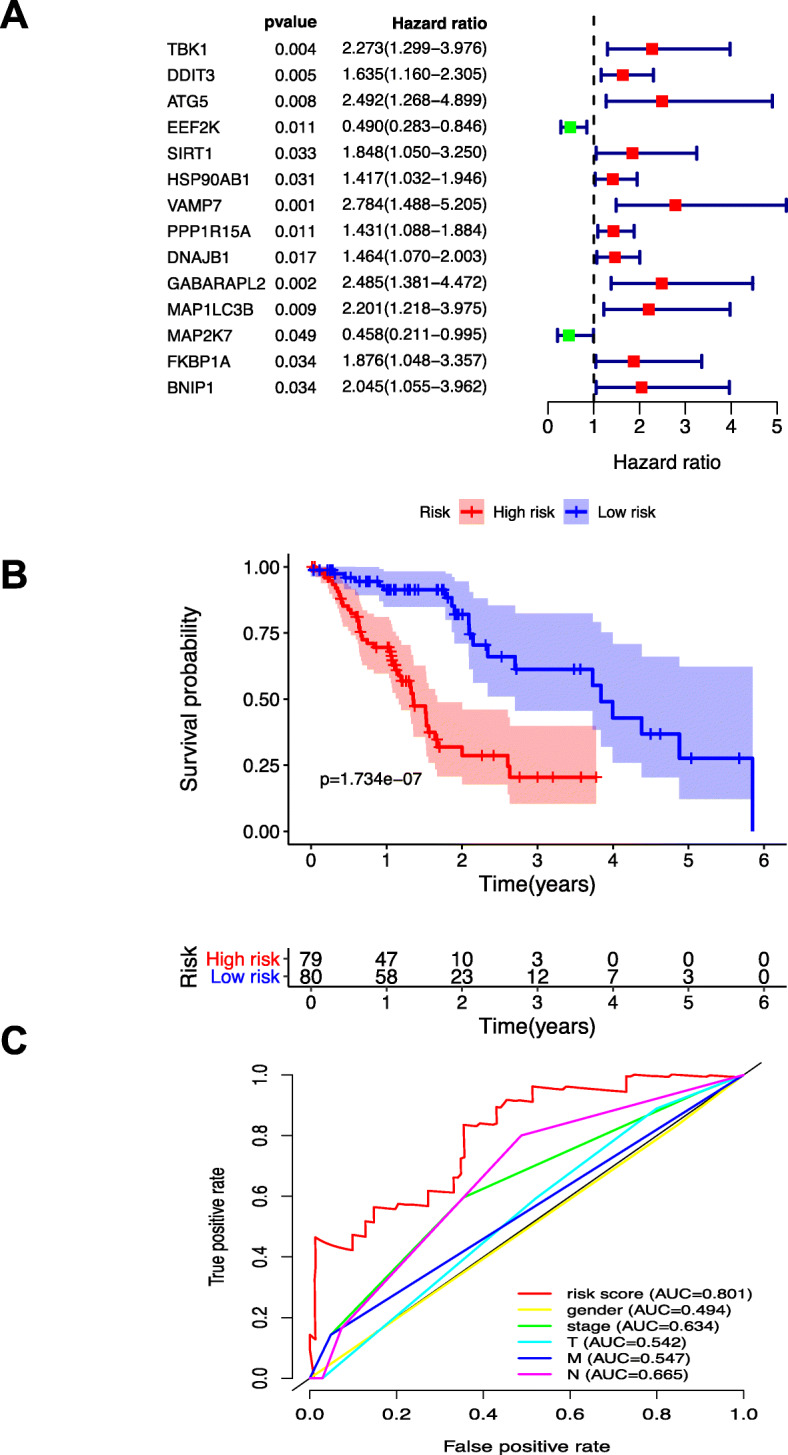
ARGs within patients with esophageal carcinoma were used to construct and analyze the risk models for OS. **a** ARGs expression among esophageal carcinoma cases was analyzed through univariate Cox regression analysis **b** OS for esophageal carcinoma cases with low (green line) and high (red line) risk was analyzed by Kaplan-Meier survival curve. **c** ROC curves showing the values of AUC for OS among esophageal carcinoma cases

Univariate Cox regression analysis was performed to examine ARGs expression within esophageal carcinoma cases, for the sake of identifying the significance of ARGs in prognosis prediction. According to our results, TBK1, ATG5, HSP90AB1, VAMP7, DNAJB1, GABARAPL2, and MAP2K7 expression was remarkably related to patient OS (*P* < 0.05). (Fig. 2a).

To identify the best signature to in prognosis prediction, multivariate Cox proportional hazards regression analysis was further carried out. As suggested by our results, TBK1 (HR 1.877, 95%CI 0.985–3.574), ATG5 (HR 2.913, 95%CI 1.464–5.795), HSP90AB1 (HR 1.449, 95%CI 1.005–2.087), VAMP7 (HR 2.712, 95%CI 1.310–5.614), DNAJB1 (HR 1.688, 95%CI 1.255–2.270), GABARAPL2 (HR 2.853, 95%CI 1.585–5.136), and MAP2K7 (HR 0.510, 95%CI 0.217–1.199) were identified as the independent adverse prognostic factors. The risk score calculating formula was (0.6295 × TBK1 expression) + (1.0691 × ATG5 expression) + (0.3706 × HSP90AB1 expression) + (0.9976 × VAMP7 expression) + (0.5236 × DNAJB1 expression) + (1.0485 × GABARAPL2 expression) - (0.6739 × MAP2K7 expression).

### Higher ARGs score was related to the worse OS

Table 1 displays the baseline characteristics of all included patients. As suggested by our log-rank test results obtained using the Kaplan-Meier survival curve, compared with low-risk cases, the high-risk cases showed poor prognosis (Fig. 2b).

**Table 1:** Baseline characteristics of patients with esophageal carcinoma

Characteristics	Variable	Total (183)	Percentages (%)
Gender	Female:	27	14.75%
	Male:	156	85.25%
Stage	Stage I	18	9.83%
	Stage II	78	42.62%
	Stage III	55	30.05%
	Stage IV	9	4.92%
	Unknown	23	12.57%
T	T0	1	0.55%
	T1	31	16.94%
	T2	43	23.50%
	T3	86	46.99%
	T4	5	2.73%
	Unknown	17	9.29%
M	M0	134	73.22%
	M1	9	4.92%
	Unknown	40	21.86%
N	N0	76	41.53%
	N1	68	37.16%
	N2	12	6.56%
	N3	8	4.37%
	Unknown	19	10.38%
Survival rate	Survival	74	40.44%
	Death	109	59.56%

**Fig. 3:**
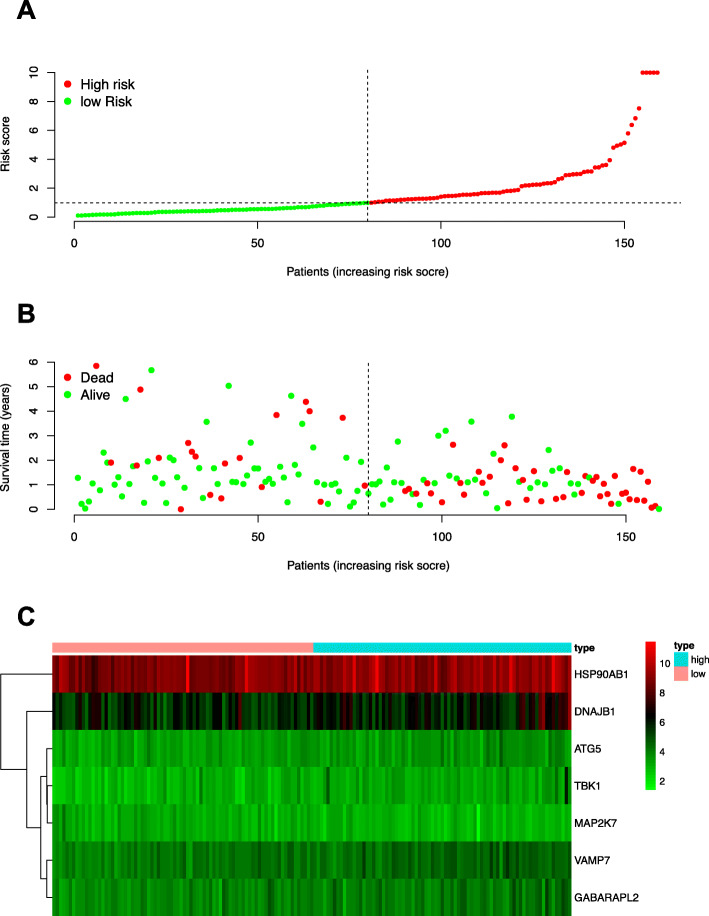
Prognosis for cases with low and high risk esophageal carcinoma. **a** Distribution of risk scores for esophageal carcinoma cases in OS model with low (green) and high (red) risks. **b** Scatter plot showing esophageal carcinoma case survival status within that OS model. Green and red dots represent the survival and death of patients, respectively. **c** Risk gene expression in low (pink) and high (blue) risk esophageal carcinoma cases within that OS model

Then, the ROC curves were plotted to determine the effect of that as-constructed signature on predicting patient OS. As suggested by our results, the value of AUC was 0.801 for those prognostic models to predict OS, which was greater than that of gender (AUC = 0.494), stage (AUC = 0.634), T (AUC = 0.542), N (AUC = 0.665), and M (AUC = 0.547). Afterwards, the distributions of patient OS risk scores were ranked and examined (Fig. 3). To better predict the clinical outcomes for patients with esophageal carcinoma, baseline characteristics (including gender, tumor, node, and metastasis stage in every patient) were incorporated into ROC curve analysis (Fig. 2c).

### Gene Ontology (GO) functional enrichment analysis

**Fig. 4:**
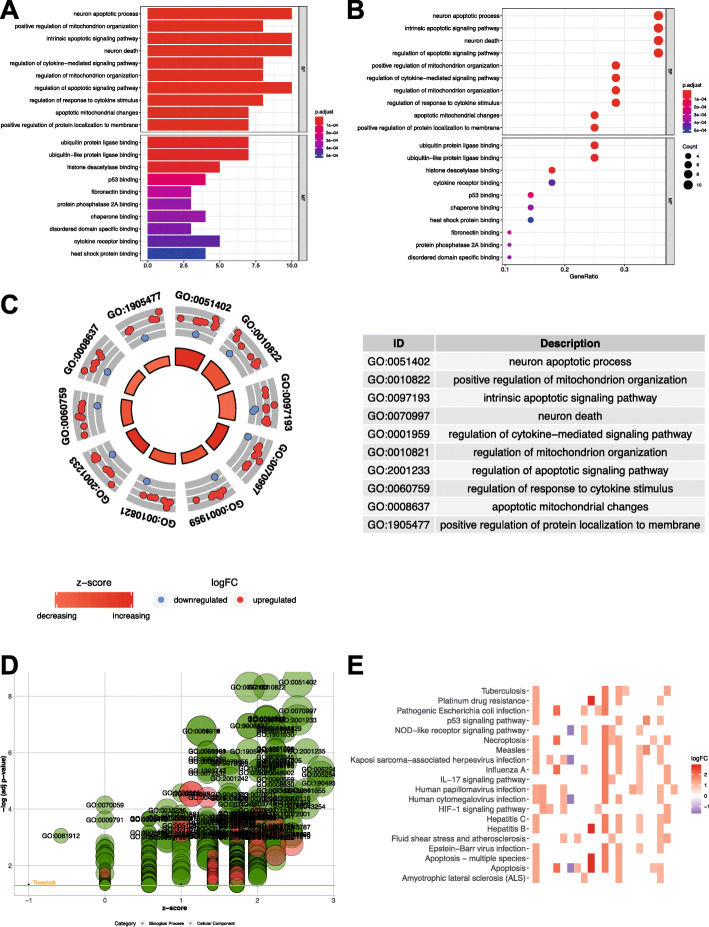
GO enrichment analysis

The above-mentioned genes with prognostic significance were extracted for functional enrichment analysis. According to the top 10 most significant GO terms with regard to MF, CC and BP categories, the above-mentioned ARGs were potentially related with ARGs regulation. (Fig. 4).

### Multivariate cox regression analysis on risk score together with baseline clinical features

**Fig. 5:**
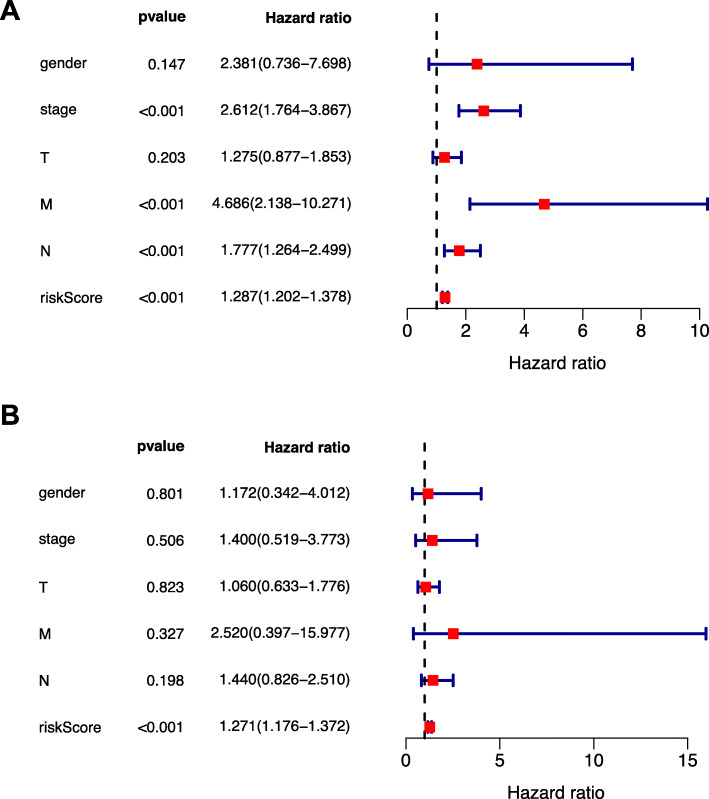
Multivariate and univariate Cox regression analyses for OS among esophageal carcinoma cases. **a-b** univariate and multivariate cox regression analyses, respectively

As suggested by the findings, only risk score (HR 1.271 95%CI 1.176–1.372) was the independent risk factor for the worse OS. (Fig. 5) Fig. 5a indicated the results of univariate cox regression analysis, and Fig. 5b showed the results of multivariate cox regression analysis.

### Associations among the risk scores, ARGs, and clinical status (including survival, gender, age, stage, T, N, and M stage)

**Fig. 6:**
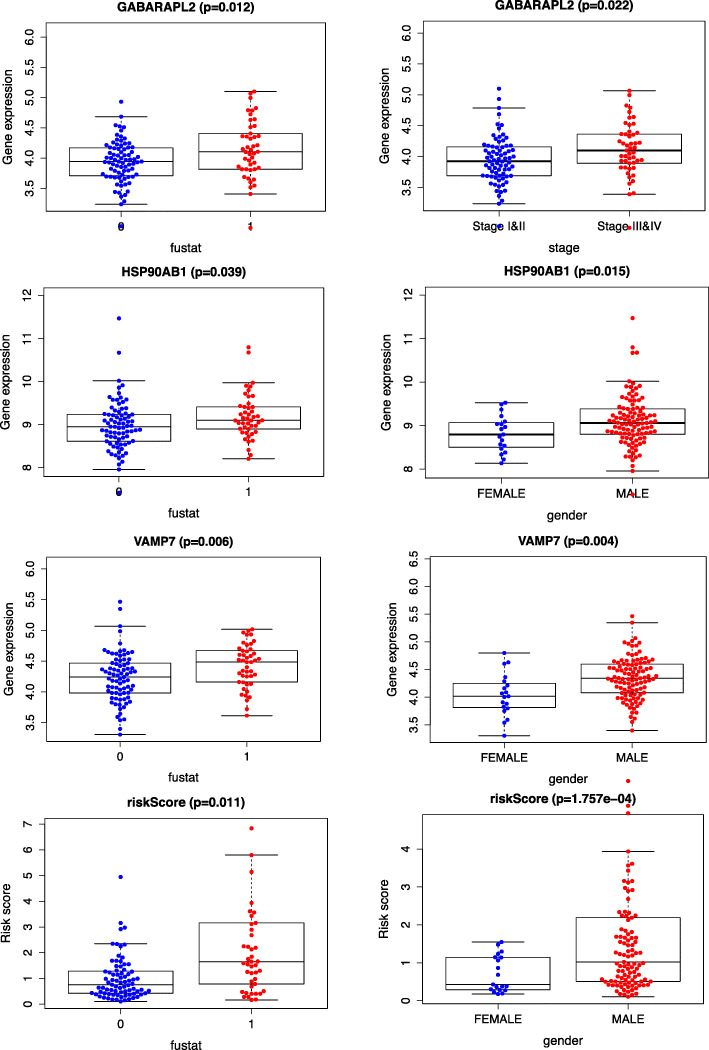
Clinical correlations among ARGs included in the risk score, risk score, and baseline clinical characteristics

The results indicated that HSP90AB1, VAMP7, and risk score were related to the survival status and gender. In addition, GABARAPL2 was markedly associated with the survival status and stage. (Fig. 6).

### The online database Oncolnc (http://www.oncolnc.org/) was used to test the included ARGs

**Fig. 7:**
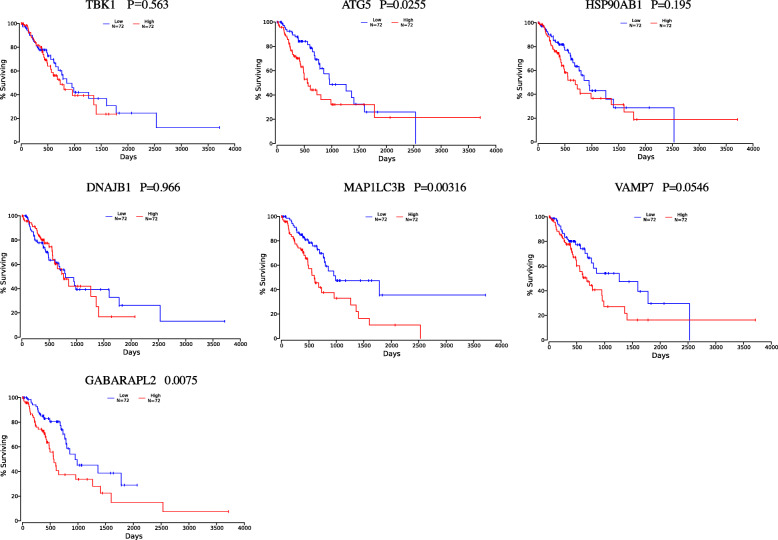
Verification of ARGs included in the risk score by Oncolnc (http://www.oncolnc.org/)

The high-risk ARGs were correlated with poor prognosis; however, the low-risk DEARGs showed significant association with favorable patient prognosis. (Fig. 7).

## Discussion

Histologically, esophageal carcinoma can be classified as adenocarcinoma and squamous cell carcinoma [24]. This study included both esophageal cancer subtypes to develop a more helpful tool for predicting the prognosis for esophageal carcinoma. Obviously, the risk score calculated by ARGs demonstrated superiority to other classical clinical indicators, and it was also the independent risk factor for patient survival.

In physiological situation, autophagy, which serves as a crucial catabolic process, works as an intracellular quality control system to maintain internal environment homeostasis through removing the damaged proteins [25]. However, autophagy has been proven to play an important role in various disorders, including cancer, degradation, autoimmune disease and inflammation [23]. During cancer development, autophagy promotes cancer cell survival within various environments [26, 27]. As the new therapeutic approach, the mechanism of autophagy in tumor has long been proposed. Even, Sui et al. [28] pointed that autophagy was accessary for responses to chemoradiotherapy.

Firstly, our study verified the different expression of ARGs between esophageal carcinoma and normal tissues. Therefore, it may be of significant to exploit a useful autophagy-related risk score for patients with esophageal carcinoma. According to our rsult, the risk score calculated by the DEARGs score was superior to other classical clinical indicators.

Among the studies related to esophageal carcinoma, Langer et al. [23] showed that autophagy was correlated with esophageal carcinoma treatment and diagnosis. To treat esophageal carcinoma using targeting ARGs, several studies try to develop the useful treatments for esophageal carcinoma through enhancing or inhibiting autophagy. In the study conducted by Huang, et al. [29], the results showed that Pristimerin reduced the growth of esophageal carcinoma. Moreover, previous studies also indicate that ginsenoside Rk3 [30] and Sinoporphyrin sodium (DVDMs)-Photodynamic therapy (PDT) [31], which exert their functions by targeting autophagy, inhibit the survival of esophageal carcinoma cells. Furthermore, other studies also illustrate that the treatment targeting autophagy contributes to enhancing the anti-tumor effect [32–34], which functions based on the chemotherapy agents (cisplatin [33], 5-fluorouracil (5-FU) [34]).

Our results in this study showed that several genes served as the risk factors for patient prognosis. Of them, ATG5 and TBK1 had attracted our great interests. For ATG5, Cheng et al. [33] demonstrated in the esophageal carcinoma study that ATG5 was involved in autophagy activation. Additionally, Zheng et al. [35] suggested that ATG5 inhibition contributed to treatment for esophageal carcinoma patients. Furthermore, autophagy abolition through the ATG5/7 re-sensitized EC109/CDDP knockdown or the use of pharmacological inhibitors is greatly significant [36] not only in the esophageal, but also in gastric [37], colorectal [38, 39], bladder [40], ovarian [41], and prostate cancers [42]. With regard to TBK1, it has been proven that TBK1 takes part in modulating cell growth and autophagy [43]. Moreover, Sarraf et al. [44] also indicated that TBK1 exerted an important role in mitophagy.

## Conclusions

In conclusion, the major ARGs are taken to develop a novel scoring system in this study, which accurately predict the clinical outcomes for esophageal carcinoma patients. The included ARGs are confirmed using the online dataset oncolnc. Therefore, our results show that ARGs can be recognized as the therapeutic targets and prognostic biomarkers for esophageal cancer. Nonetheless, our findings should be further investigated in the future.

## Data Availability

Each dataset used in this study was searched based on the published literature, freely accessible from related authors upon request.

## Abbreviations

ARGs: Autophagy-related genes
OS: Overall survival
ROC: Receiver operating characteristic
AUC: Area under curve
TBK1: TANK-binding kinase 1
ATG5: Autophagy protein 5
HSP90AB1: Heat shock protein 90 kDa alpha, class B member 1
VAMP7: Vesicle-Associated Membrane Protein 7
DNAJB1: DnaJ homolog subfamily B member 1
GABARAPL2: Golgi-associated ATPase enhancer of 16 kDa
MAP2K7: Mitogen-activated protein kinase kinase 7
PET-CT: Positron Emission Tomography-Computed Tomography
HER2: Human Epidermal Growth Factor Receptor 2
PD-L1: Programmed Death 1
TCGA: The Cancer Genome Atla
PCA: Principal component analysi
GO: Gene Ontology
